# Practical guidance for using multiple data sources in systematic reviews and meta‐analyses (with examples from the MUDS study)

**DOI:** 10.1002/jrsm.1277

**Published:** 2017-12-15

**Authors:** Evan Mayo‐Wilson, Tianjing Li, Nicole Fusco, Kay Dickersin, for the MUDS investigators

**Affiliations:** ^1^ Department of Epidemiology Johns Hopkins University Bloomberg School of Public Health 615 North Wolfe Street Baltimore MD 21205 USA

**Keywords:** meta‐analysis, multiple data sources, reporting bias, risk of bias assessment, selective outcome reporting

## Abstract

Data for individual trials included in systematic reviews may be available in multiple sources. For example, a single trial might be reported in 2 journal articles and 3 conference abstracts. Because of differences across sources, source selection can influence the results of systematic reviews. We used our experience in the Multiple Data Sources in Systematic Reviews (MUDS) study, and evidence from previous studies, to develop practical guidance for using multiple data sources in systematic reviews.

We recommend the following: (1) Specify which sources you will use. Before beginning a systematic review, consider which sources are likely to contain the most useful data. Try to identify all relevant reports and to extract information from the most reliable sources. (2) Link individual trials with multiple sources. Write to authors to determine which sources are likely related to the same trials. Use a modified Preferred Reporting Items for Systematic Reviews and Meta‐analyses (PRISMA) flowchart to document both the selection of trials and the selection of sources. (3) Follow a prespecified protocol for extracting trial characteristics from multiple sources. Identify differences among sources, and contact study authors to resolve differences if possible. (4) Prespecify outcomes and results to examine in the review and meta‐analysis. In your protocol, describe how you will handle multiple outcomes within each domain of interest. Look for outcomes using all eligible sources. (5) Identify which data sources were included in the review. Consider whether the results might have been influenced by data sources used. (6) To reduce bias, and to reduce research waste, share the data used in your review.

## 1 INTRODUCTION

Systematic reviews and meta‐analyses of clinical trials are used to determine the effectiveness and safety of interventions, and they provide a foundation for decision making. To ensure their results are replicable, systematic reviewers aim to include all (1) trials that have been performed and are relevant to their research questions and (2) relevant data about the design, risk of bias, and results of those trials.

Data for individual trials may be available in multiple public sources (eg, journal articles, conference abstracts, trial registrations, and regulatory reviews) and nonpublic sources (eg, clinical study reports [CSRs] and individual participant data [IPD]). Accessing multiple data sources is challenging and resource intensive, in part, because all sources are not available from a single library or website. Furthermore, individual data sources are often incomplete, and multiple data sources may include contradictory data about the same trial (Box 1). Thus, source selection may influence the results of meta‐analyses included in systematic reviews.

**Box 1: Summary box**

- Multiple data sources are available for most clinical trials, which may include the same or different information about trial design, risk of bias, and results.
- Systematic reviews rarely describe how they handle multiple sources for a single trial, and there is little existing guidance about this problem.
- Systematic reviewers should prespecify which sources will be used for data extraction and analysis, indicate the *main* source for each trial in the review, and identify when information in the review comes from another source.
- Systematic reviewers should share the data and the sources used for analysis to prevent duplication of effort and to reduce research waste.

**Figure 1:**
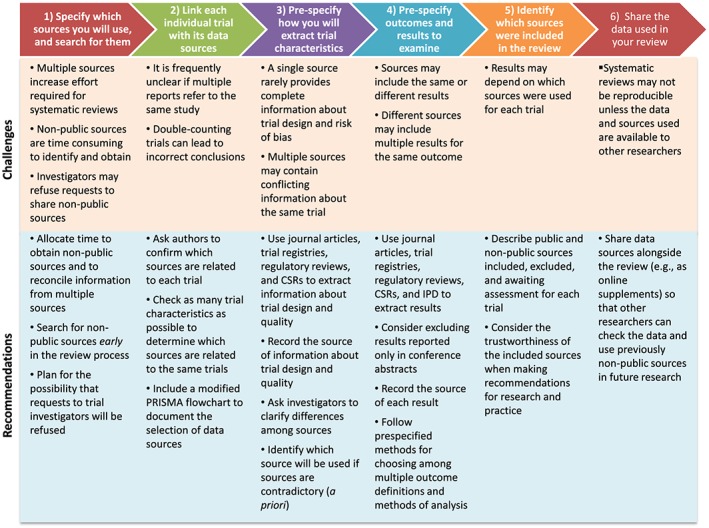
Evidence‐based recommendations to address the challenges of using multiple sources in systematic reviews [Colour figure can be viewed at http://wileyonlinelibrary.com]

This article provides practical guidance for using multiple data sources in systematic reviews and meta‐analyses. This guidance was developed by consensus among the authors based on our experience in the Multiple Data Sources in Systematic Reviews (MUDS) study and based on previous studies comparing multiple data sources (Figure 1).

Throughout the paper, we provide examples from the MUDS study. As described elsewhere, the MUDS study was an investigation pertaining to the methodology of systematic reviews (*not* a systematic review) that followed a published protocol for 2 case studies: (1) gabapentin for neuropathic pain and (2) quetiapine for bipolar depression.^1^ We believe this was the first study to compare information that could be obtained using *all* available data sources for systematic reviews. We found that trials included many outcomes and results, which created opportunities for selective reporting by both trialists and systematic reviewers.^2^ By including information from multiple data sources selectively, we found that a systematic reviewer could manipulate the results and interpretation of individual trials and meta‐analyses.^3^

In this guidance, we focus on existing data sources (eg, reports and databases). Systematic reviewers sometimes contact trialists to ask questions about study design or to request specific results, and these issues have been considered elsewhere.^4^, ^5^ In this guidance, we do not provide detailed recommendations for communicating with trialists. Although the MUDS study included 2 pharmaceutical interventions, our findings were consistent with evidence from previous studies that included other types of interventions; additional studies of behavioral, procedural, device, and other trials are needed. Some of the sources described in this guidance are available only for medical products (eg, CSRs), and additional sources may be available for systematic reviews of nonpharmacological interventions (eg, treatment manuals for trials of complex interventions).

## 2 STEP 1. SPECIFY WHICH SOURCES YOU WILL USE FOR YOUR REVIEW AND SEARCH FOR ELIGIBLE SOURCES

Authors of high‐quality systematic reviews conduct comprehensive searches and often find multiple data sources for eligible trials; this step is resource intensive, and some data sources are more useful than others (Table 1). On the basis of our own experience, and guidance from Cochrane and the National Academy of Medicine,^6^, ^7^ we recommend that every systematic review team includes an informationist who is trained and knowledgeable about searching for multiple data sources.

**Table 1:** Strengths and limitations of different sources

	Source	Strengths	Limitations
Public sources	Journal articles	Found easily Extracted quickly Include useful data about methods and results	Available for some, but not all studies Contain limited study characteristics and methods Omit outcomes that can be found in nonpublic sources (especially harms)
	Short reports (eg, conference abstracts)	Identify otherwise unpublished studies	Include little information about trial design and risk of bias Often misleading (eg, report incorrect number of groups and sample size) May result in double‐counting trials in meta‐analysis
	Trial registrations	Identify otherwise unpublished trials May contain information about design, risk of bias, and results not included in other public sources Link multiple sources about the same trial using unique registration numbers	Limited to new studies that comply with registration requirements Often contain limited information about trial design and results Report only harms (adverse events) occurring above a threshold (eg, 5%) Outdated for trials that have not updated their methods and results
	Regulatory information	Identify trials not reported in other public sources Describe methods and results not found in other sources	Available only for trials submitted to regulators (eg, trials conducted before marketing authorization) Available for approved indications, but not *off‐label* uses
Nonpublic sources	Clinical study reports (CSRs)	Contain detailed information about study characteristics, methods, and results (including harms) Describe aggregate results, which are easy to analyze and sufficient for most reviews	Missing or difficult to obtain for most trials May use outdated methods for analysis (eg, methods for handling missing data) May not address questions of interest for the review (limited to investigators' analyses) Require more time to obtain and analyze than public sources
	Individual patient data (IPD)	Allow reviewers to use contemporary statistical methods and to standardize analyses across trials Permit analyses not included in the original trial reports (eg, subgroup analyses)	Require considerable expertise and time to obtain and analyze May lead to the same results that can be found in aggregate report (if analyzed using the same methods)

Before beginning a systematic review, you should consider which sources may be available and which sources may contain the most useful data for your review. For example, while most systematic reviews will search for journal articles reporting the methods and results of relevant trials, questions about drug effectiveness and safety may be especially likely to be associated with both public and nonpublic data sources. In the MUDS study, for example, we identified journal articles for most trials, and we obtained CSRs for most of the trials conducted by industry; however, we did not find the same data sources for all trials (Table 2). Clinical study reports are documents used by drug and device manufacturers for summarizing trial methods and findings.^8^ Like previous studies,^9^ we found that CSRs included much more information than journal articles. In the MUDS study, CSRs were available for the early trials conducted by drug manufacturers; however, CSRs were not available for studies conducted by independent researchers. As in the MUDS study, understanding what sources of information are likely to be available for each trial in your review will help you determine what to look for and how to search efficiently.

**Table 2:** Data sources for trials included in the Multiple Data Sources in Systematic Reviews study (adapted from Mayo‐Wilson et al^2^)

	Gabapentin	Quetiapine
Number of trials	21	7
Sources of data for each trial (no. of trials, % of all trials)		
Only public	15 (71%)	3 (43%)
Only nonpublic	1 (5%)	0 (0%)
Both public and nonpublic	5 (24%)	4 (57%)
Trials with each source type (no. of trials, % of all trials)		
IPD	6 (29%)	1 (14%)
Journal article about 1 trial	17 (81%)	6 (86%)
Journal article about ≥2 trials	7 (33%)	4 (57%)
Short report: conference abstract	10 (48%)	6 (86%)
Short report: other	9 (43%)	4 (57%)
Trial registration	5 (24%)	7 (100%)
FDA report	2 (10%)	0 (0%)
CSR synopsis	0 (0%)	2 (29%)
CSR	6 (29%)	2 (29%)

Abbreviations: CSR, clinical study report; FDA, Food and Drug Administration; IPD, individual participant data.

Public sources include journal articles, conference abstracts, FDA reviews, trial registrations, and other reports (letters to the editor, posters, press releases, and reports in trade publications).

Nonpublic sources include CSRs, CSR synopses, and IPD.

### 2.1 Identify public data sources

Journal articles are the most important public data source for most systematic reviews. Even when multiple data sources are available, systematic reviewers often use only journal articles or use them as the *main* source of data. In addition to being readily available, we found that data in journal articles were usually consistent with data in nonpublic sources.^2^ Thus, we recommend identifying all journal articles relevant to your research question, and we recommend using them in conjunction with other sources to describe the methods and results of eligible trials.

Short reports (eg, conference abstracts) are commonly available for trials, but on the basis of MUDS findings, we believe they have limited utility. While short reports may identify trials that are not reported elsewhere,^10^, ^11^ there is little evidence that they contain usable data for systematic reviews. In the MUDS study, most conference abstracts did not include sufficient results information to include them in meta‐analyses. For example, many *P* values were reported without magnitude or precision of the effects (eg, “All five pain scores improved by Week 10 for patients who received G‐QD (p = 0.046–0.002).”)^12^ In the MUDS study, many short reports claimed that gabapentin “was generally well tolerated” but did not describe how harms were selected for inclusion in the report and did not include numerators and denominators necessary for meta‐analysis of harms; for example, one described only percentages for “dizziness (10.9% vs. 2.2% for placebo) and somnolence (4.5% vs. 2.7% for placebo).”^12^ Previous studies also found that short reports often overstate the benefits of interventions and underreport their harms (adverse events).^10^, ^13^ Thus, we recommend that you use short reports to identify eligible trials for your systematic review; however, we recommend you do not use them as the primary source to assess risk of bias or to extract data for meta‐analyses. If you decide to use short reports to assess risk of bias or to extract data for meta‐analyses, we recommend that you conduct sensitivity analyses by excluding short reports to identify their impact on the results.

Trial registries (eg, ClinicalTrials.gov) are useful for identifying trials, comparing published results with planned outcomes and methods and obtaining results not reported elsewhere.^11^, ^14^, ^15^ Registries may also include data about potential harms that cannot be obtained through other sources.^16^ The utility of trial registrations could be improved if a greater proportion of clinical trials were registered,^17^, ^18^ accurate, and up‐to‐date and if results were posted for a greater proportion of trials.^17^, ^18^, ^19^, ^20^ Trials that report data about harms in registers often report only those events occurring above a certain quantitative threshold (eg, 5% of participants),^21^, ^22^ which could contribute to incorrect conclusions in systematic reviews and meta‐analyses. We recommend that you search trial registries for eligible trials and compare data in trial registries with data in other sources (eg, journal articles).

Regulatory reviews (eg, US Food and Drug Administration [FDA] and European Medicines Agency) include data about trials of drugs, biologics, and medical devices submitted by manufacturers for marketing approval.^23^ They sometimes include data about trials not reported in journal articles.^24^, ^25^ Studies comparing regulatory reviews with journal articles about antipsychotics and antidepressants found that some trials considered *negative* or *inconclusive* by regulators were reported as *positive* in journal articles.^26^, ^27^ In the MUDS study, we found that regulatory reviews may not be a good source of information about harms for use in meta‐analysis. The FDA Medical Review^28^ and Statistical Review^29^ of gabapentin for postherpetic neuralgia (available from Drugs@FDA) both reported pooled data about harms, but neither included the results for each individual trial. Additionally, regulatory reviews do not contain data about trials conducted after marketing approval. We recommend you check regulatory reviews when conducting a systematic review on a regulated product (eg, drugs, biologics, and devices) to determine whether they contain trials not reported elsewhere.

Trial protocols may be available in multiple locations. For example, the United States requires that protocols and statistical analysis plans be submitted with results on ClinicalTrials.gov for trials funded by the National Institutes of Health^30^, ^31^ and for trials of drugs, biologics, and medical devices.^21^, ^22^ When protocols are not public, they may be available from certain journals, trial funders, as appendices in CSRs, or by contacting the investigators.

### 2.2 Identify nonpublic data sources

Clinical study reports are submitted to regulators with trial datasets, and they typically contain the protocol and statistical analysis plan as appendices and present aggregated data, including detailed analyses that take account of missing data. They often contain more data about trial methods and results (ie, benefits and harms) than any other single data source.^32^ In the MUDS study, the length of CSRs ranged from 1315 to 8027 pages,^2^ a finding consistent with previous studies.^9^

Although much information must be extracted from CSRs by systematic reviewers, computer programs may be helpful for extracting data from lengthy tables. In the MUDS study, we used ABBYY FineReader software (version 11) to extract IPD and data about harms that were included as tables in appendices.^2^ Although CSRs provide the most complete aggregated data about harms,^33^ most systematic reviews of harms do not use them.^34^ This may be because, despite their advantages, obtaining CSRs can be challenging. While they are currently available upon request from the European Medicines Agency for drugs submitted for approval, the FDA does not make CSRs publicly available.^35^ Many CSRs are available through unsealed litigation documents and other sources.^36^, ^37^ We recommend that you use CSRs when possible, even when trials have been reported in public sources (eg, journal articles), and that you search for and request CSRs from manufacturers for all trials of drugs, biologics, and medical devices.

Individual participant data have been described as the *gold standard* for systematic reviews.^7^, ^38^ Our experience in the MUDS study suggests that reanalyzing IPD may not be necessary if one has a CSR. Reanalyzing IPD requires more resources and expertise compared with reviewing aggregated data, including time from statisticians and senior investigators. In the MUDS study, gabapentin IPD were in electronic databases, and quetiapine IPD were in tables as appendices to a CSR, and we converted the data to an electronic format; IPD were not accompanied by metadata in either case.^3^ Furthermore, some systems used to record data in older trials are not in current use. In the MUDS study, older trials used the Coding Symbols for a Thesaurus of Adverse Reaction Terms system, which was last updated in 1999 to record harms; newer trials used the Medical Dictionary for Regulatory Activities system, which has been used since the 1990s (http://www.meddra.org). Reanalyzing IPD may be useful, however, when you cannot locate the data you seek in a CSR, when you wish to apply new or different methods of analysis (eg, handling of missing data), or when you wish to examine subgroups that are not described in other sources.

Searching for nonpublic sources is resource intensive, regardless of whether requests are fulfilled or refused.^39^ Thus, we recommend that you begin searching repositories (eg, http://www.clinicalstudydatarequest.com) and contacting trialists (eg, academic investigators and drug and device manufacturers) as early as possible to negotiate data use agreements and to gain access to data. When both CSRs and IPD are available, we recommend using CSRs unless you need to reanalyze IPD to answer your research questions.

## 3 STEP 2. LINK EACH INDIVIDUAL TRIAL WITH ITS DATA SOURCES

Linking multiple sources for individual trials is important to ensure that trials are not double‐counted.^40^, ^41^ This may be relatively easy for recent trials with identifiers (eg, trial registration numbers). However, some sources do not include identifiers, and other sources include incorrect identifiers,^42^, ^43^ so methods for linking sources are difficult to automate.^44^ Sometimes multiple sources about the same trial do not reference each other, do not share common authors,^40^, ^41^ do not include enough information about their design and results to determine whether they describe the same trial, or include different information about the same trial.^3^ In addition, some sources are undated; in these cases, it may be difficult to determine whether the sources are related to the same trial and which data are most current.

We recommend that you write to authors to confirm the sources associated with each trial. For example, one manufacturer declined to provide CSRs and IPD for the MUDS study^39^ but did provide a list of publications and presentations, which allowed us to confirm how those reports were related to the included trials. When you are unable to confirm the relationships among sources by contacting authors, you should check as many trial characteristics as possible to determine which sources are most likely related to the same trials.

**Figure 2:**
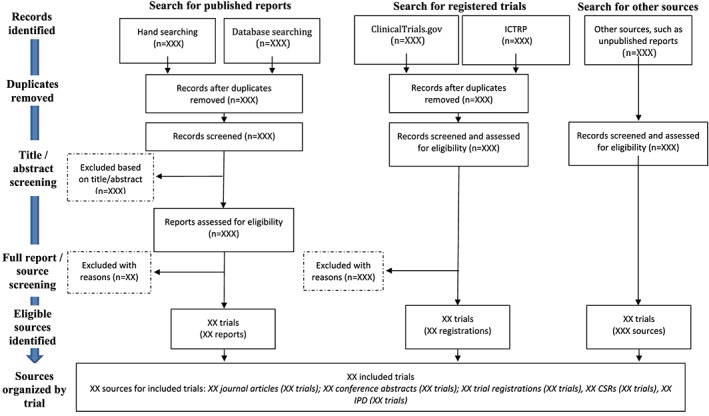
Modified Preferred Reporting Items for Systematic Reviews and Meta‐analyses (PRISMA) flowchart to describe multiple sources [Colour figure can be viewed at http://wileyonlinelibrary.com]

To document both the selection of trials and the selection of sources, we recommend that you include a modified Preferred Reporting Items for Systematic Reviews and Meta‐analyses^45^ (PRISMA) flowchart as part of the systematic review (Figure 2).

## 4 STEP 3. PRESPECIFY HOW YOU WILL EXTRACT TRIAL CHARACTERISTICS FROM MULTIPLE SOURCES

The protocol for a systematic review should describe how you will work with multiple data sources for each individual trial. Once you have identified all sources for each eligible trial, you will extract data from them and enter those data in a database in which each record represents either (1) a single eligible trial (ie, data from all sources will be included in the same record) or (2) an eligible data source for each eligible trial (ie, there will be multiple records related to each eligible trial).

If you decide that each record in your database will represent a single trial, you should identify the *main* data source for each trial. Because you may not know ahead of time which data sources will be available, it may be impossible to prespecify the main source for each trial. You can anticipate this in your protocol by prespecifying which source will be used when sources include conflicting data and these differences cannot be resolved by contacting authors. When data from multiple sources will be entered into a single record, we recommend that you identify which data were extracted from a source other than the *main* source and identify conflicting data in different sources.

## 5 STEP 4. PRESPECIFY OUTCOMES AND RESULTS TO EXAMINE IN THE SYSTEMATIC REVIEW AND META‐ANALYSIS

When multiple sources are used, multiple syntheses containing many outcomes may be possible.^3^ In the MUDS study, as in previous studies,^46^, ^47^ it was possible to change the results of meta‐analyses by selectively choosing information from multiple data sources. For example, we could change the overall magnitude and statistical significance of the effect of gabapentin on pain intensity by selecting the largest (overall standardized mean difference = −0.45; 95% confidence interval −0.63 to −0.27) or smallest treatment effect (standardized mean difference = −0.06; 95% confidence interval −0.24 to 0.12) reported across multiple data sources.^2^ To prevent bias and to limit unnecessary work, you should prespecify in the systematic review protocol the outcomes and associated results for each outcome (ie, numerical estimates of treatment effects) that you will extract for your meta‐analysis.^2^, ^46^, ^47^

Outcomes in systematic reviews are often described at the level of outcome domains,^47^ such as *depression*; however, a complete outcome definition includes the outcome domain (eg, depression), specific measurement (eg, Beck Depression Inventory), specific metric (eg, change from baseline), method of aggregation (eg, continuous), and time point.^47^, ^48^ By varying these elements (eg, using multiple measures of depression), and by using multiple methods of analysis (eg, varying the method of handling missing data), a single trial can include dozens of results related to the same outcome domain.^49^ Because you are unlikely to use information about all outcomes and results in each trial, we recommend you prespecify how you will prioritize outcomes for data extraction and analysis within each outcome domain of interest (Box 2). In the MUDS study, we found that many trial reports did not define all elements of each outcome, even if they included enough results information to include them in a meta‐analysis; you should prespecify how to will handle outcomes that are not defined clearly.

**Box 2: Prespecify the elements of each outcome you will examine in your systematic review and meta‐analysis**

**Outcome:** In your systematic review, you should define each *outcome* you will examine.^2^, ^50^ In both trials and systematic reviews, you may find that *outcomes* are referred to by *titles*, for example, *pain intensity*. This title, however, is just one of the 5 elements that define each outcome and is called the *outcome domain*. The 5 elements that define an outcome are outcome domain, time point, specific measure, specific metric, and method of aggregation.^48^

**Outcome domain:** The outcome domain is the title^47^ or concept^51^ to describe one or more outcomes

**Time point:** A single trial might assess the same outcome domain more than once, and different trials might assess the same outcome domain at different time points. You should prespecify how you will handle multiple time points within and across trials; for example, you might combine outcomes for meta‐analysis if they were assessed within a *time window* such as *8 weeks (4 to 12 weeks)*.

**Specific measure:** Trials often include multiple specific measures (ie, scales or instruments) related to a single domain. You should prespecify and provide a rationale for (1) combining different measures across trials in a single meta‐analysis (eg, using the standardized mean difference) or (2) conducting separate analyses for different measures. If you plan to combine multiple specific measures for meta‐analysis, you should prespecify how you will select or calculate a single result from each study to include in the analysis.

**Specific metric:** Trials often include multiple units of measurement (eg, value at a time point, change from baseline, and time‐to‐event). You should prespecify how you will handle multiple specific metrics within and across trials, including whether you will combine results for multiple metrics and, if applicable, how you will address statistical problems that arise from combining different specific metrics in the same meta‐analysis.^7^

**Method of aggregation:** Multiple methods of aggregation might lead to multiple results for the same specific measure (eg, mean change, proportion with 50% improvement). You should specify which analyses will include continuous, categorical, and time‐to‐event variables. Within each of those broad categories, you should prespecify how you will handle multiple methods of aggregation both within individual trials and across multiple trials (eg, 30% improvement, 50% improvement).

We recommend prespecifying in the review protocol that outcomes will be extracted using all eligible sources rather than limiting extraction to the outcomes and results reported in the main source. To limit the time required for data extraction and reconciliation, we suggest that you extract only those outcomes and the associated results that will be included in meta‐analyses.

## 6 STEP 5. IDENTIFY WHICH SOURCES WERE INCLUDED IN THE REVIEW

In the MUDS study, we found that interventions could appear more or less beneficial depending on the data source. For example, interventions for which CSRs or IPD are available might appear to be less beneficial than interventions reported in public sources only. You should consider how the availability and selection of data sources might have affected your results, particularly if you are comparing several interventions for which different types of data sources are available.

Using multiple sources can help you identify reporting biases. For example, it might be clear from a journal article that reports a dichotomous outcome (eg, the proportion of participants achieving a threshold value at 8 weeks) that accessing IPD would allow you to analyze the original outcome data using continuous information (eg, the mean change from baseline at 8 weeks). In addition to extracting information from multiple sources, you may use multiple sources of a single trial to identify known gaps and to determine whether further information about the included trials could affect your conclusions.

## 7 STEP 6. SHARE THE DATA USED IN YOUR REVIEW

Systematic reviewers should work with trialists, journal editors, funders, regulators, and other stakeholders to make trial data publicly available. We encourage systematic reviewers to share the data used in systematic reviews where it would be legal and ethical to do so (eg, all participant identifiers have been removed from IPD). Sharing data sources, and sharing the data used for analysis, allows verification and reduces waste because data will not have to be collected again for future updates of the review.

## 8 CONCLUSION

Identifying multiple data sources for a single trial creates a number of challenges and opportunities. Systematic reviewers should think through and prespecify methods for selecting and synthesizing information from multiple data sources; any amendments to what was planned should be publicly documented and dated. Because journal articles are not available for all trials, and journal articles do not include all of the information that can be found in other sources,^52^, ^53^ systematic reviewers also should examine trial registries, regulatory reviews, and CSRs to extract information about trial design, risk of bias, and results. Individual participant data may be useful for some meta‐analyses in systematic reviews, but IPD meta‐analyses are not always necessary to assess effectiveness and safety. There have been many discussions about the use of IPD (eg, Coady et al,^54^ Strom et al,^55^ and Taichman et al^56^), yet the MUDS study and previous studies demonstrate that other data sources (eg, CSRs) may be equally or more useful for many systematic reviews.^57^ To reduce bias, and to reduce research waste, systematic reviewers should share the data sources used in their reviews.

## CONFLICT OF INTEREST

Up to 2008, Kay Dickersin served as an unpaid expert witness for the plaintiffs' lawyers in litigation against Pfizer that provided several gabapentin documents used for the MUDS study. Swaroop Vedula was paid by the plaintiffs' attorneys for research assistance provided to Kay Dickersin for her work as an expert witness. Greene LLP, a law firm that has represented various clients in litigation against Pfizer, including the case above in which Kay Dickersin served as an expert witness, provided a fund for scholarly research on reporting biases that partially covered salaries of some investigators performing analysis and interpretation of data.

## AUTHOR CONTRIBUTION

The study design was first described in the application to the Patient‐Centered Outcomes Research Institute (PCORI) in 2013. Kay Dickersin was the principal investigator and worked with Tianjing Li and Peter Doshi to design the study, write the application, and obtain the funding. Evan Mayo‐Wilson drafted the protocol with contributions from other authors.

All authors contributed to interpretation and data presentation. Evan Mayo‐Wilson wrote the first draft of the manuscript. All authors reviewed, provided critical revisions, and approved the manuscript for publication.

Evan Mayo‐Wilson is the guarantor. All authors, external and internal, had full access to all of the data (including statistical reports and tables) in the study and can take responsibility for the integrity of the data and the accuracy of the data analysis.

## ETHICS APPROVAL

The MUDS study received an exemption from the Johns Hopkins Bloomberg School of Public Health Institutional Review Board (IRB no. 00006324).

## TRANSPARENCY DECLARATION

Evan Mayo‐Wilson affirms that the manuscript is an honest, accurate, and transparent account of the study being reported; that no important aspects of the study have been omitted; and that any discrepancies from the study as planned (and, if relevant, registered) have been explained.
